# The cardioprotective effect of danshen and gegen decoction on rat hearts and cardiomyocytes with post-ischemia reperfusion injury

**DOI:** 10.1186/1472-6882-12-249

**Published:** 2012-12-10

**Authors:** Fan Hu, Chi-Man Koon, Judy Yuet-Wa Chan, Kit-Man Lau, Kwok-Pui Fung

**Affiliations:** 1 School of Biotechnology and Food Engineering, Hefei University of Technology, Hefei, Anhui, PR China; 2 State Key Laboratory of Phytochemistry & Plant Resources in West China, The Chinese University of Hong Kong, Shatin, New Territories, Hong Kong; 3 Institute of Chinese Medicine, The Chinese University of Hong Kong, Shatin, New Territories, Hong Kong; 4 School of Biomedical Sciences, The Chinese University of Hong Kong, Shatin, New Territories, Hong Kong; 5 509A, Lo Kwee-Seong Integrated Biomedical Sciences Building, Area 39, The Chinese University of Hong Kong, Shatin, New Territories, Hong Kong SAR, China

**Keywords:** Danshen and Gegen decoction, Ischemia and reperfusion, Hypoxia and reoxygenation, Apoptosis, Calcium

## Abstract

**Background:**

Danshen (Salviae Miltiorrhizae Radix) and Gegen (Puerariae Lobatae Radix) have been used for treating heart disease for several thousand years in China. It has been found that a Danshen and Gegen decoction (DG) exhibiting an anti-atherosclerosis effect, which improves the patients’ heart function recovery. Pre-treatment with DG was reported to have protective effects on myocardium against ischemia/reperfusion injury. In the present study, we aim to investigate the post-treatment effect of DG on ischemic-reperfusion injuries *ex vivo* or *in vitro* and the underlying mechanisms involved.

**Methods:**

The rat heart function in an ischemia and reperfusion (I/R) model was explored by examining three parameters including contractile force, coronary flow rate and the release of heart specific enzymes within the heart perfusate. *In vitro* model of hypoxia and reoxygenation (H/R), the protective effect of DG on damaged cardiomyocytes was investigated by examining the cell structure integrity, the apoptosis and the functionality of mitochondria.

**Results:**

Our results showed that DG significantly improved rat heart function after I/R challenge and suppressed the release of enzymes by damaged heart muscles in a dose-dependent manner. DG also significantly inhibited the death of cardiomyocytes, H9c2 cells, with a H/R challenge. It obviously decreased cell apoptosis, protected the mitochondrial function and cell membrane skeleton integrity on H9c2 cells. The cardio-protection was also found to be related to a decrease in intracellular calcium accumulation within H9c2 cells after I/R challenge.

**Conclusion:**

The potential application of DG in treating rat hearts with an I/R injury has been implied in this study. Our results suggested that DG decoction could act as an anti-apoptotic and anti-ion stunning agent to protect hearts against an I/R injury.

## Background

The myocardial tissue is typically aerobic and its metabolism is closely dependent upon oxygen availability which is confirmed by the abundance of mitochondria and myoglobin in the cardiomyocytes [1]. In addition, the high-energy requirement of cardiomyocytes for its contraction is met almost exclusively by mitochondrial oxidative phosphorylation [2]. This, in turn, leads to the high sensitivity of myocardial cells to oxygen deficiency. Once the blood flow to the myocardial tissue is blocked and the oxygen shortage environment (ischemia) is subsequently induced, serious cardiac damage will happen in the ischemic region for the decrease of energy supply and acidosis inducing by anaerobic glycolysis [3,4], the notable decrease of intracellular pH value and the increase of intracellular calcium ion concentration ([Ca^2+^_i_) [5-7]. Clinically, there are obviously increasing release of heart damage enzyme markers (creatine kinase and lactate dehydrogenase) in the serum of patients with myocardial ischemic injury [8]. Additionally, the decrease contractile force or ventricular akinesis for the myocardium damage disturbs the normal blood circulation, which intensifies the heart tissue injury [1,9]. In this case, a series of cardiac injury events will happen, such as a decrease in the energy-supplying substances (such as ATP, phocereatin and glycogen), an increase in lactate production and acidosis for anaerobic glycolysis [6,7], an accumulation of the substrates for generating superoxide (O_2_^-^) and hydrogen peroxide, a notable decrease of the intracellular pH value and an increase of intracellular calcium ion concentration ([Ca^2+^_i_) [5-7]. Although the restoration of blood supply in the ischemic region is essential for salvage of myocardium, the accompanying reperfusion during the first few minutes can induce worse effect on myocardium rather than improve its dysfunction [10]. This procedure is called post-ischemic reperfusion injury, which is characterized by the sharp production of superoxide and other reactive oxygen species (ROS), extreme exacerbation of the ionic disturbances (such as, Ca^2+^) in the cytosol and mitochondria. These can induce peroxidation of membrane lipid, denaturation of the proteins on ion channels and cytoskeleton proteins (such as, Troponin I, which is also a myo-contractile factor) (including enzymes and ion channels) as well as the breaking-down of DNA [8,11,12]. Increased production of ROS and the accumulation of calcium in the cytosol and mitochondria are considered to be two major causative factors involving this injury [13,14]. They also could induce the damage of mitochondria (such as, the opening of the mitochondrial permeability transition pore, mPTP), which then triggers itself depolarization, more mitochondrial ROS generation, and intense cell demisemore cytochrome c release from mitochondria into cytosol [15,16]. Those damage-related factors can directly initiate the myocardium apoptosis pathway to intense cell dysfunction and demise [12,16].

Traditional Chinese medicine (TCM) has the benefit of multi-targeting and synergism, which features have been widely used in China to treat heart diseases. Among them, Danshen and Gegen are commonly present in herbal formulae for treating human heart diseases [17]. Chemical Danshen is the dried root of *Salviae miltiorrhizae* and it can be divided into lipophilic and hydrophilic fractions. The main active components of its hydrophilic fraction are salvianolic acid B, danshensu, protocatechuic acid, and rosmarinic acid [18-20]. Some research groups have shown that the water extraction of Danshen decreased the infarct size and protected the mitochondrial membrane function in rat hearts which had an I/R injury [21-23]. Gegen is the dried root of *Pueraria lobata*. The chemical constituents of Gegen are mainly isoflavonoids, such as puerarin, diadzin, daidzein, 8-C-apiosylglucoside, genistin and genistein [24,25]. The components puerarin, daidzin and diadzein have been found to exert cardiovascular protection by inhibiting platelet aggregation and decrease of serum cholesterol [26-28].

In our previous studies, a Danshen and Gegen decoction (DG) in the ratio of 7 to 3 (w/w) was found to exert anti-atherosclerotic property to protect cardiovascular system both in clinical and pre-clinical studies [29,30]. Especially, pretreatment of DG inhibited the oxidation stress in the rat heart with ischemia/reperfusion injury by increasing the activity of PKC pathway and improved the heart function recovery [31,32]. In the present study, and I/R rat heart model and an hypoxia and reperfusion (H/R) cellular model have been applied to examine whether DG exert a cardiac protective effect against ischemic reperfusion injury. Our findings could provide evidence that DG would be a useful decoction for treating patients who are undergoing cardiac surgery and thrombolytic therapy which are usually accompanied with ischemic reperfusion injury.

## Methods

### Herbal extract and chemicals

The raw herbs of Danshen and Gegen were purchased from the herbal shops in Sichuan and Guangdong province, respectively. The herbal extraction was carried out by National Engineering Research Center for Modernization of Traditional Chinese Medicine, a GMP compliance manufacturer in the People’s Republic of China. The quantification of the major components in DG decoction was conducted in accordance with our previous study [33]. A small quantity of the two raw herbs were deposited as voucher specimen in the museum of the Institute of Chinese Medicine in The Chinese University of Hong Kong, with voucher specimen number of 2008-3166a and 2008-3167a for Danshen and Gegen, respectively. Same batch of DG decoction was used throughout the experiment in our present study. Unless otherwise specified, the chemicals used in this study were purchased from Sigma-Aldrich Chemical Company. KB-R 7943 was purchased from Tocris Bioscience (USA).

### Cell culture

H9c2 cells are an adherent rat cardiac cell line with myoblast morphology. These cells were obtained from American Tissue Culture Collection (Manassas, VA) and cultured in DMEM with 10% FBS and 1% PS.

### Ischemia and reperfusion Langendorff heart model

220–250 g Male Sprague–Dawley (SD) rats were supplied by Laboratory Animal Sciences Center (LASEC) of The Chinese University of Hong Kong (CUHK). All experimental protocols were approved by the Animal Experimental Ethics Committee (AEEC) at the CUHK. The rats were killed by cervical dislocation and their hearts were excised within one minute thereafter. The hearts were then put into cold heparinized Kreb’s solution (4.7 mM KCl, 2.5 mM CaCl_2_, 1.2 mM MgSO_4_, 1 mM KH_2_PO_4_, 11 mM glucose, 25 mM NaHCO_3_ at pH 7.4) with 50 unit/ml heparin to prevent blood clotting. The surrounding connective tissues and fats were removed. The aorta of each rat heart was then cannulated on a Langendorff apparatus. The heart was then perfused with Kreb’s solution (gassed with 95% O_2_, 5% CO_2_) and enclosed in a 37°C pre-warmed organ bath. A basal force of 2 g was then applied to these rat hearts. After equlibrated for 20 min, the supply of Kreb’s solution was stopped for 30 min (ischemia). At the last minute of ischemia, 200 μl of DG solution which contained 0.4 mg or 1.6 mg of DG extract (dissolved in Kreb’s solution) or trolox (20 μM) [34] were injected into the rat heart through its aorta before reperfusion. Reperfusion was then started and lasted for 15 min. Kreb’s solution was used instead of DG for the control group. Various parameters were measured at 10th min and 20th min during the equilibration as the normalizing parameter and every minute during the 15-min reperfusion.

### Measurement of contractile force

The contractile force was determined by a wire which was connected to a force- displacement transducer. The force-displacement transducer (Power lab/8sp, AD Instruments) was connected to a Powerlab and the force was translated into a digital signal. The recording rate was set at 1 K/s. The percentage contractile force recovery was the contractile force in every minute during reperfusion when compared to the average values at 10th min and 20th min of equilibration.

### Measurement of coronary flow rate

The amount of the coronary heart perfusate was collected and measured at 1 min interval for 15 min. It was assumed that the density of the heart perfusate was about 1 g/ml (which is the same as water). The weight of the heart perfusate collected in 1 min was the average flow rate (ml/min). The percentage coronary flow rate recovery was the coronary flow rate in each minute during reperfusion as compared to the average values at 10th min and 20th min of equilibration.

### Measurement of creatine kinase (CK) and lactate dehydrogenase (LDH) activity

Coronary heart perfusate were placed on ice after weighing until they were assayed for creatine kinase (CK) and lactate dehydrogenase (LDH) activity. The extent of CK and LDH leakage during the reperfusion period, as an indication of myocardial injury, was estimated by the percentage of CK and LDH accumulative activity at each minute of the reperfusion time (1–15 minutes). In the present study, the CK and LDH content were measured by commercially available kits (Stanbio Laboratory, USA). For measuring CK activity, 20 μl of the heart perfusate were added to 30°C pre-warmed cuvette containing 1 ml CK reagent and mixed by inversion. After 3 min incubation, the absorbance was read at 340 nm, versus water as a reference. Absorbance was measured at 30-second intervals for a period of 120 seconds. The activity of CK in U/l is the change of absorbance per minute (ΔA/min).

For LDH measurement, 50 μl of the heart perfusate was added to 30°C pre-warmed cuvette containing 1 ml CK reagent and mixed by inversion. After 3 min incubation, the absorbance was read at 340 nm versus water as a reference. Absorbance was read at 10-second intervals for a period of 60 seconds. The activity of LDH in U/l is the change of absorbance per minute (ΔA/min).

### Hypoxia and reoxygenation cardiomyocytes model

Hypoxia and reoxygenation (H/R) on H9c2 cells were performed as described with minor modifications [35,36]. After seeding the cells overnight, the culture medium was replaced by a salt balance solution for 1 hour to synchronize the cells in each group. Hypoxia medium (bubbled with N_2_ for 30 min) was then added to replace the salt balance solution. The plate was then put into a hypoxia chamber and incubated at 37°C with the oxygen removed. After 12 hours incubation, the hypoxia medium was substituted by normal medium with or without DG and the cells were incubated for another 4 hours before performing various assays.

### Determination of the viability of H9c2 cells in a normoxic situation with a H/R injury

H9c2 cells (6 × 10^3^/well) were seeded on 96-well plate and incubated overnight. In normoxic environment, the cells were treated with DG for 24 hours. In H/R injury situation, the cells were firstly challenged with hypoxia for 12 h and were then treated with or without DG (125 μg/ml and 250 μg/ml) or trolox (20 μM as positive control) for 4 hours in a reoxygenation phase [34]. Cell viability was then measured by MTT assay by adding 30 μl of MTT solution (5 mg/ml) to each well, which were then incubated at 37°C, 5% CO_2_ humidified incubator for 4 h. After that, 50 μl of dimethyl sulfoxide (DMSO) was added to dissolve the crystals. Absorbance at 540 nm was finally measured by using a microplate reader.

### Determination of cell apoptosis with Annexin V-FITC and PI double staining

H9c2 cells (3 × 10^5^/well) were seeded in a 6-well plate and were then incubated overnight. After hypoxia and reoxygenation treatment, the cells were collected by trypsization, washed with PBS and then resuspended in 250 μl of binding buffer (10 mM HEPES, 150 mM NaCl, 5 mM KCl, 1 mM MgCl_2_, 2 mM CaCl_2_, pH 7.4). A 250 μl reaction binding buffer containing Annexin V-FITC PI was then added to the resuspended cells. The cells were kept on ice throughout the experiment. The mixture was kept at room temperature in the dark. After 15 min incubation, cell apoptosis was analyzed by FACSCanto flow cytometer (Becton Dickinson).

### Measurement of mitochondrial depolarization

H9c2 cells (3 × 10^5^/well) were seeded in 6-well plate and incubated overnight. After hypoxia and reoxygenation treatment, the cells were collected by trypsization, washed with PBS and then resuspended in JC-1 solution (10 μM in PBS). The cells were then incubated in 37°C for 15 min in dark and were finally analyzed by a FACSCanto flow cytometer (Becton Dickinson).

### Measurement of calcium localization in H9c2 cells

H9c2 cells were seeded into a confocal culture dish (3 × 10^5^). After overnight incutbation, the cells were then incubated with an ischemia medium containing Fluo-4 AM solution (5 μM in PBS). After 12 h, the ischemia medium was replaced by Fluo-3 medium (140 mM NaCl, 5 mM KCl, 1 mM CaCl_2_, 1 mM MgCl_2_, 10 mM glucose, 10 mM HEPES, pH 7.4) with or without DG (125 μg/ml and 250 μg/ml) or KB-R 7943 (10 μM as positive control) [7]. Calcium signal was measured for 15 min under confocal microscope (Olympus, Japan) with an excitation wavelength of 488 nm and a light emission of 520 nm.

### Western blotting analysis

Total cellular protein was used for detecting caspase 3, bcl 2 and Troponin I expressions. Mitochondrial and cytosolic proteins were used for detecting the distribution of cytochrome c in these cellular fractions. An equal amount of protein (30 μg) was resolved by 8% or 12% SDS-polyacrylamide electrophoresis gel. The resolved proteins were transferred to a PDVF-membrane. After blocking with 5% non-fat milk for 60 min, the blots were incubated overninght at 4°C with respective antibodies: anti-caspase 3 (1:1000, Cell Signaling, USA, Cat # 9662), anti-bcl 2 (1:1000, Cell Signaling, USA, Cat # 2870), anti-Tropinin I (1:1000, Cell Signaling, USA, Cat # 4002), anti-cytochrome c (1:1000, Cell Signaling, USA, Cat # 4272), COX-IV (1:1000, Cell Signaling, USA, Cat # 4844) and β-actin (1:10000, Sigma-Aldrich, USA, Cat # A2228). Anti-rabbit HRP conjugated IgG (1:1000, Invitrogen, USA, Cat # 65–6120) and anti-mouse HRP-conjugated IgG (1:1000, Invitrogen, USA, Cat # 62–6520) were used to detect the binding of their corresponding antibodies by incubating for 2 h at room temperature. The resolved protein was then visualized by developing the image on a film using ECL Western Blotting Detection Reagents (GE Healthcare, UK).

### Statistical analysis

The results were expressed as a mean ± S.E.M. The statistical differences between groups were analyzed by One-way or Two-way ANOVA followed by a Bonferroni post-hoc test. All tests were carried out at 5% level of significance (two-tailed, p *<* 0.05).

## Results

### Effects of DG decoction on cardiac function recovery and damage-related enzyme release

**Figure 1:**
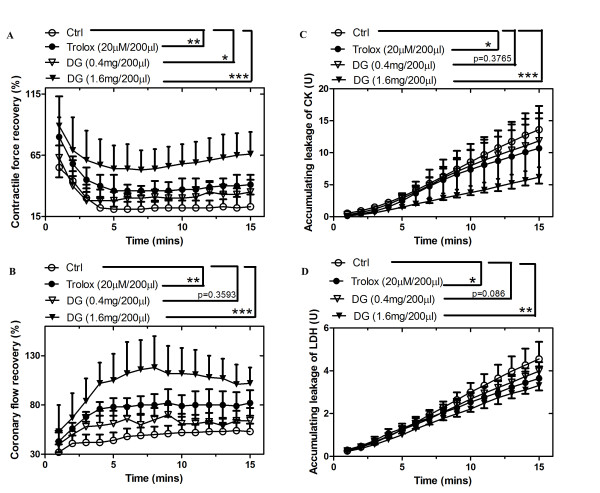
**The effects of DG on cardiac function recovery and damage-related enzyme release.A**) The contractile force recovery of an ischemia reperfusion-injured isolated rat hearts was protected by DG. The force recovery of a rat heart in each time point for a rat heart is the ratio of the contractile force at indicated time point to the force before ischemia; **B**) The coronary flow rate recovery of an ischemia reperfusion-injured isolated rat hearts was protected by DG. The flow recovery in each time point is the ratio of the flow rate at indicated time point to the rate before ischemia; **C**) The accumulating leakage of creatine kinase (CK) of isolated rat hearts was inhibited by DG; **D**) The accumulating leakage of lactate dehydrogenase (LDH) of isolated rat hearts was inhibited by DG. Curves were plotted by the mean of 6 rat hearts per group (n = 6). The data were expressed as mean ± S.E.M. *p < 0.05, **p < 0.01 and ***p < 0.001 indicated the groups with significant difference when compared to the control group.

In Figure 1A, low dosage of DG (0.4 mg/200 μl) showed a small but significant improvement on the contractile force recovery when compared to the control group. The contractile force recovery percentage was 35% and 23% in low dosage of DG and in control group, respectively. Obvious protection was shown when the dosage of DG was increased to 1.6 mg/200 μl, where the recovery at the last minute of reperfusion was 66%.

As shown in Figure 1B, 2 mg/ml of DG improved the coronary flow rate without a significant difference as compared to the control group. The recovery percentages at the end point was 64% and 53%, respectively. At high dosage of DG, the recovery was noticeably increased to 102% at the end of the reperfusion. A similar effect was observed in the positive control of trolox, which significantly improved the coronary flow rate to 82%.

As shown in Figure 1C, low dosage of DG did not show any obvious protection against CK leakage (11.86 U/l), which was similar to that in the control group (13.61 U/l). However, there was significant protection against CK leakage at a high dosage of DG (6.17 U/l), when compared to that in control group. Trolox had similar protection as high dosage group which reduced the CK release significantly.

Figure 1D showed LDH release in each group. Low dosage DG group did not show significant protection against LDH release (3.98 U/l), which was similar to that in control group (4.55 U/l). At high dosage, there was a significant reduction of LDH release (3.32 U/l) when compared to that in control group. LDH release for the trolox-treated group (3.64 U/l) was similar to that of high dosage DG group.

### Effect of DG decoction on H9c2 cell survival after a H/R challenge

**Figure 2:**
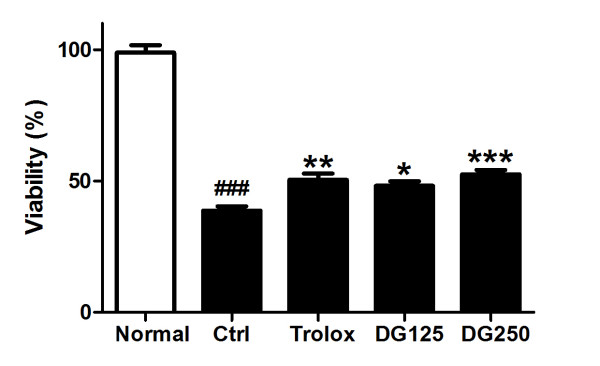
**The protection of DG against hypoxia and reoxygenation (H/R)-induced injury in H9c2 cells.** After 12 h of hypoxia, the solution was changed into a normal medium with or without DG (125 and 250 μg/ml) or trolox (20 μM) in the 4 hours reoxygenation period. Cell viability was then measured by MTT assay. The data were expressed as mean ± S.E.M. Six replicates were done in each experiment. Three independent experiments were performed. *p < 0.05, **p < 0.01 and ***p < 0.001 indicated the groups with significant difference when compared to the control group; ### p < 0.001 when compared to the normoxic group.

The survival of H9c2 cells after a H/R-challenge was assessed by MTT assay (Figure 2). It was found that H/R treatment significantly reduced cell viability to 38.74% when compared to the normal control (no H/R treatment). The viability of H9c2 cells with H/R-challenge was significantly increased to 48.19% and 54.92% at 125 and 250 μg/ml DG treatment, respectively (p < 0.05 and p < 0.001). The effect of DG was similar to that of trolox which increased cell viability to 50.46% (p < 0.01).

### Effect of DG decoction on membrane skeleton integrity of H9c2 cells

**Figure 3:**
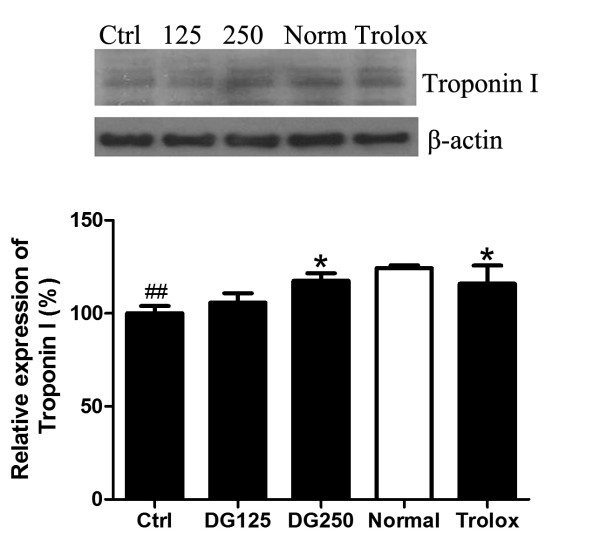
**The effect of DG on Troponin I protein expression in H9c2 cells.** After H9c2 cells were challenged with H/R in the presence or absence of DG (125 and 250 μg/ml) or trolox (20 μM), the expression of Troponin I was analyzed by Western blot and was normalized by β-actin. The figure is a representative of three individual experiments. The data were expressed as mean ± S.E.M. *p < 0.05 and **p < 0.01 indicated the groups with significant difference when compared to the control group; ### p < 0.001 when compared to the normoxic group.

To explore the cell membrane skeleton integrity, Troponin I expression was analyzed by Western blotting. As shown in Figure 3, the relative amount of the Troponin I in DG-treated cells (250 μg/ml) was upregulated from 100% to 117.5% when compared to control. The relative amount of Troponin I was 124.3% in a normoxia group and 115.9% in trolox-treated cells with a H/R challenge (p < 0.01 and p < 0.05, respectively).

### Effect of DG decoction on apoptosis induction in H9c2 cells after a H/R challenge

**Figure 4:**
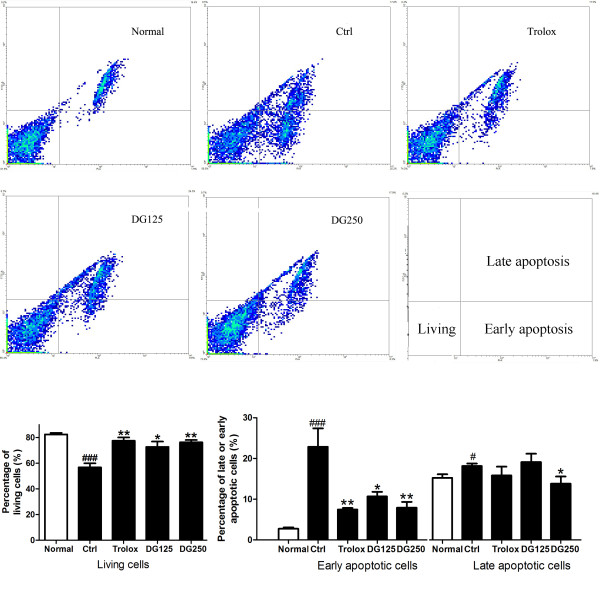
**The effect of DG on H/R-induced apoptosis in H9c2 cells.** After H9c2 cells were challenged by H/R in the presence or absence of DG (125 and 250 μg/ml) or trolox (20 μM), apoptotic cells were analyzed by Annexin V/PI staining. The data were expressed as mean ± S.E.M. Three independent experiments were performed. ^#^p < 0.05 and ^###^p < 0.001 indicated the groups with significant difference when compared to the normoxic group. *p < 0.05 and **p < 0.01 indicated the groups with significant difference when compared to the control group.

As shown in Figure 4, DG significantly inhibited the early apoptosis of H9c2 cells which was induced by a H/R challenge and the percentage of early apoptotic cells number was reduced from 22.87% (control) to 9.73% and 10.67% in the 125 μg/ml and 250 μg/ml DG-treated group, respectively (p < 0.05 and p < 0.01). The percentage of early apoptotic cells in normaxic and trolox-treated group were 2.37% and 7.4%, respectively (p < 0.001and p < 0.01). High concentration of DG (250 μg/ml) was also found to significantly decrease the late apoptosis of H9c2 cells with H/R injury (p < 0.05).

**Figure 5:**
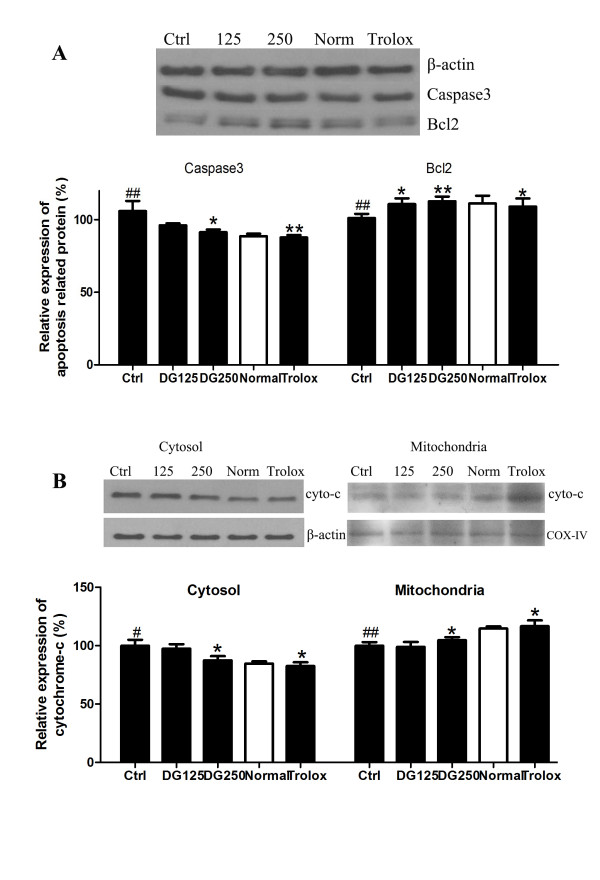
**The effect of DG on apoptotic protein expression in H/R-challenged H9c2 cells.** After treatment with H/R in the presence or absence of DG, **A**) Caspase 3 and bcl 2 expression in total cellular protein extract was detected by Western blot which was normalized by β-actin. **B**) Cytochrome c expression in cytosolic fraction (normalized by β-actin) and mitochondrial fraction (normalized by COX-IV) were detected by Western blot. Relative band intensity was calculated by dividing the intensity of each treated sample by that of the control group. The data were expressed as mean ± S.E.M. The figure is a representative of three individual experiments. The data were expressed as mean ± S.E.M. #p < 0.05 and ##p < 0.01 indicated the groups with significant difference when compared to the normoxic group. *p < 0.05 and **p < 0.01 when compared to the control group.

To further investigate the underlying mechanism, the expression level of caspase 3 and bcl 2 were investigated. As shown in Figure 5A, the relative expression of caspase 3 was significantly decreased to 91.3% in 250 μg/ml DG treated-group as compared to the control (set as 100%). Bcl 2 expression was increased at both DG concentrations (125 and 250 μg/ml) by about 10% (p < 0.05 and p < 0.01, respectively), which was similar to the normaxic group and the trolox-treated group (p < 0.01 and p < 0.05).

### Effect of DG decoction on cytochrome c release from mitochondria of damaged H9c2 cells

Figure 5B showed that cytochrome c release from mitochondria to cytosol was decreased in a dose-dependent manner in DG-treated groups. For cytosolic fraction, similar to the positive control, the relative amount of cytochrome c was 87.5% in DG (250 μg/ml) treated group (p < 0.05). On the other hand, in mitochondria, the value of cytochrome c expression was significantly increased by about 7% and 16% in high concentration of DG-treated group and trolox-treated groups, respectively (p < 0.05).

### Effect of DG decoction on mitochondrial depolarization in H9c2 cells after H/R challenge

**Figure 6:**
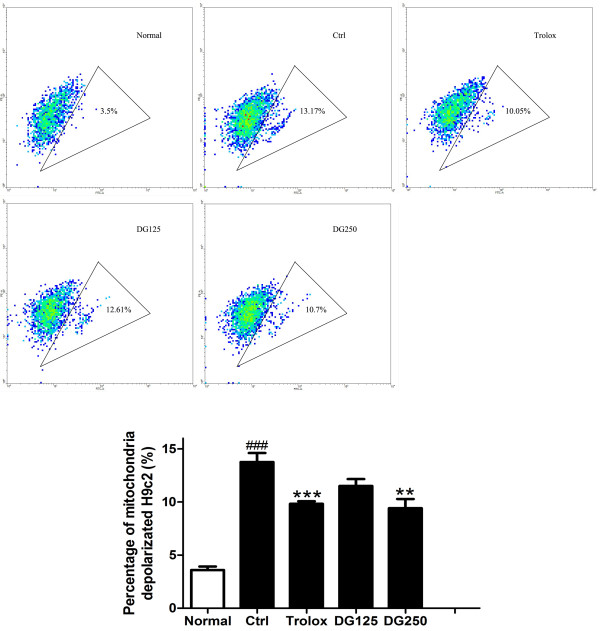
**The effect of DG on mitochondrial depolarization in H/R-challenged H9c2 cells.** After H/R was challenged in the presence or absence of DG (125 and 250 μg/ml) or trolox (20 μM), mitochondrial depolorization was measured by flow cytometry with JC-1 staining. The data were expressed as mean ± S.E.M. Three independent experiments were performed. ^###^p < 0.001 indicated the groups with significant difference when compared to the normoxic group. **p < 0.01 and ***p < 0.001 when compared to the control group.

As shown in Figure 6, H/R treatment induced the depolarization of mitochondrial membrane potential of H9c2 cells from 3.6% (normal) to 13.77% (control). Mitochondria depolarization significantly decreased to 9.43% and 8.26% in 125 μg/ml and 250 μg/ml DG-treated groups, respectively (p < 0.05 and p < 0.01) and to 9.84% in trolox-treated group (p < 0.001).

### Effect on calcium accumulation within H9c2 cells after a H/R challenge

**Figure 7:**
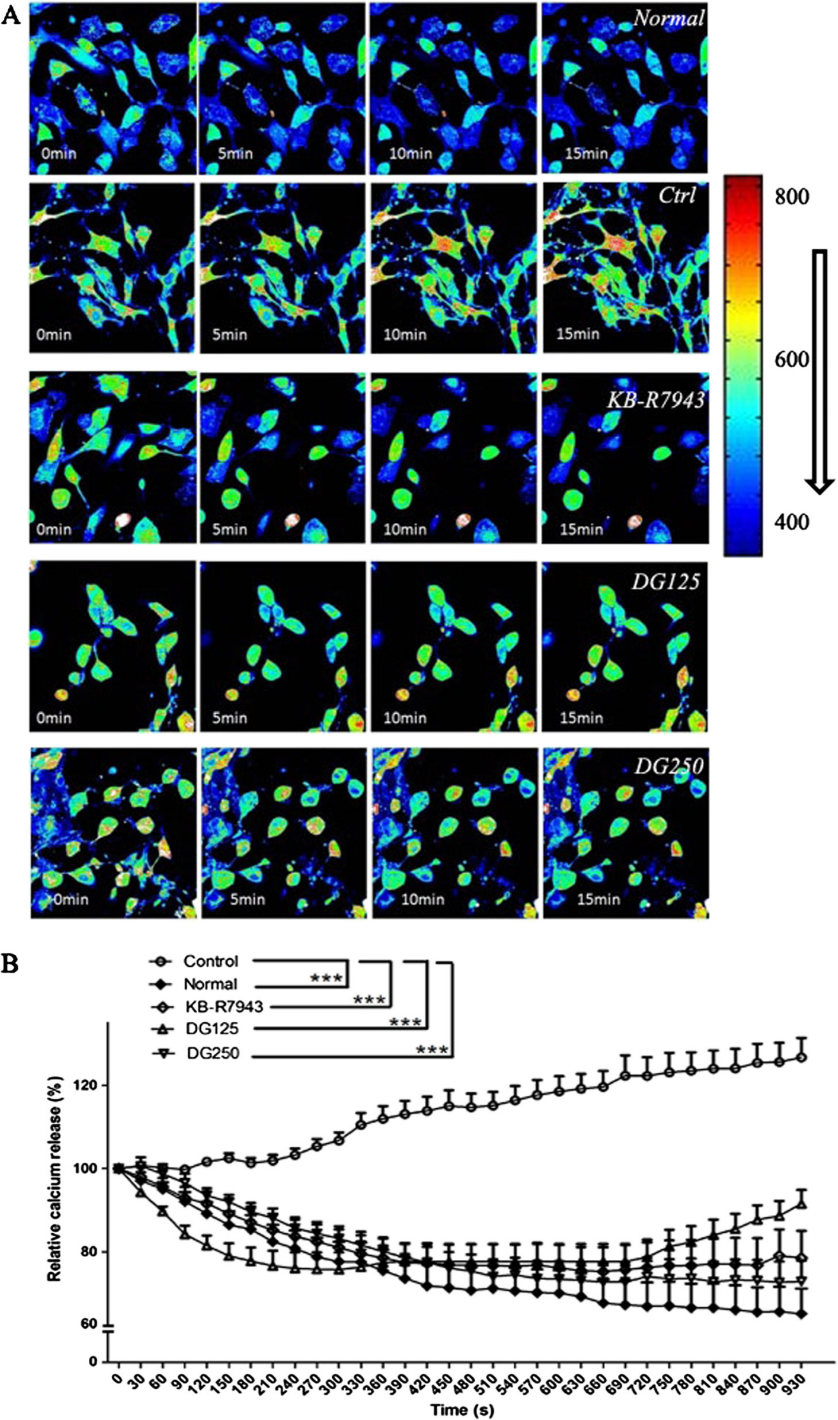
**The effect of DG on calcium accumulation induced by a H/R-challenge in H9c2 cells.A**) calcium signal was measured at 0, 5, 10 and 15 min by a confocal microscope after Fluo-4 AM staining; **B**) Quantitative, real-time measurement of calcium accumulation. KB-R 7943 was a Na^+^-Ca^2+^ exchanger antagonist, which was used as a positive control. The calcium signal was normalized to that at 0 min in each group. The data were expressed as mean ± S.E.M. Three independent experiments were performed. ***p < 0.001 indicated the groups with significant difference when compared to the control group.

Figure 7A showed a representative figure on accumulative calcium signal. It was shown that the calcium signal was induced in H9c2 cells with H/R challenge and the increase was suppressed by DG treatment or KB-R 7943 treatment (positive control). The relative accumulated calcium in normal H9c2 cells was 66.15%. After H/R-challenge, DG treatment significantly attenuated the intracellular calcium accumulation from 126.7% (control) to 91.61% and 72.89% in 125 μg/ml and 250 μg/ml DG-treated groups, respectively (p < 0.001) and to 78.55% in KB-R7943- treated groups (p < 0.001) (Figure 7B).

## Discussion

DG has been reported to have a wide range of pharmacological properties, such as vasodilation, anti-atherosclerosis and an anti-oxidative effect [29-33,37,38]. Most particularly, DG pretreatment induced cardioprotection against an I/R injury in rats by enhancing the mitochondrial antioxidant status by activating PKCε to inhibit the opening of K_ATP_ in mitochondria [31,32]. Our present study aims to explore whether the administering of DG post-treatment in the reperfusion/reoxygenation phase can directly provide protective effects against reperfusion injury *ex vivo* and *in vitro*. It was found that DG could protect the rat heart damage in the reperfusion phase by inhibiting the release of heart specific enzymes and restoring the contractile force as well as coronary flow rate recovery. In addition, DG directly and rapidly attenuated intracellular Ca^2+^ accumulation within H9c2 cells in the reoxygenation phase. DG also could inhibit the apoptosis of H9c2 cells. Our results provided evidence that there is a potential for patients with coronary heart diseases to attenuate the I/R injury in their blood restore surgeries by DG.

In our previous study, the contents of seven components, namely danshensu, protocatechuic aldehyde, puerarin, daidzein 8-capiosyl-glucoside, daidzin, salvianolic acid B and daidzein were quantified in DG water extract. Since only water extract was used, the valuable lipophilic tanshinones (cryptotanshinone, tanshinone I and tanshinone IIA) were not present in the DG extract or only in traces. Therefore, tanshinones did not contribute but water soluble marker such as salvianolic acid B might contribute to the cardiovascular protective activities [39].

In the *ex vivo* study, the recovery of coronary flow rate and of the contractile force in the reperfusion phase was examined. Oxygen delivery in the heart is dependent on two crucial factors, namely, the blood flow rate and the arterial oxygen content [40]. Flow rate recovery could be helpful for restoring oxygen and nutrient supply and in the recovery of the heart function. In the present study, DG post-treatment obviously improved the coronary flow rate and contractile force recovery after an I/R challenge in a dose-dependent manner. In the high dosage DG treatment group, the contractile force recovery of the damaged heart was up to 60% (Figure 1A) and the coronary flow rate was almost totally recovered (Figure 1B). DG treatment significantly inhibited CK and LDH release in the hearts (Figure 1C &1D). It reflected that heart damage status had been partly reversed and was consistent with the results of contractile force and coronary flow rate recovery.

An I/R challenge always results in cardiac dysfunction and cellular injury through inducing a calcium overload and destroying the contractile apparatus (Troponin I, a membrane skeleton protein) [41,42]. In the cellular model, it was found that membrane integrity of cardiomyocytes was protected by DG decoction by improving the expression of Troponin I (Figure 3). It underlines that DG post-treatment could restore the heart contraction function after an I/R challenge, by protecting the cell structure and the contractile unit integrity of cardiomyocytes. Additionally, DG treatment improved the survival of H9c2 cells after a H/R challenge (Figure 2). Figure 4 showed that the number of early apoptotic cells was obviously decreased after DG treatment. A high concentration of DG also significantly inhibited the late apoptosis of H9c2 cells_._ Inhibition of apoptosis was also examined by measuring the expression of pro-apoptosis and anti-apoptosis protein. The result showed that the expression of caspase 3 (pro-apoptotic factor) was significantly decreased and bcl 2 (anti-apoptotic factor) was significantly increased with DG treatment (Figure 5A). The increased survival ability of cardiomyocytes could improve the heart function recovery after an I/R challenge.

Over recent years, mitochondria have become a major research focus for experimental cardiologists. The exploration of the potential role of mitochondria in the pathogenesis of cardiovascular diseases, particularly in an I/R injury, has been greatly increased [43-45]. The opening of mPTP in the outer-membrane of mitochondria is a key pathological phenomenon involving in an I/R injury [46]. One of its downstream events is the rupture of the outer mitochondrial membrane, which could become permeable to molecules smaller than 1.5 kDa, and which then would induce the release of cytochrome c [47]. It is well known that when cyotochrome c is released from mitochondria, this could couple with Apaf-1, forming a macromolecular complex which recruits and activates the death effector caspase 9 [48]. The activated caspase 9 then activates caspase 3 and caspase 7, which in turn activates the early apoptosis process [49]. To further investigate the molecular mechanism of anti-apoptotic activity of DG, the nature of the cytochrome c release after a H/R challenge was determined. Figure 5B showed that DG suppressed the release of cytochrome c from mitochondria to cytosol by comparing its expression between cytosol and mitochondria. The other event is the opening mPTP which causes mitochondria to become depolarized and loses its Δψ. This event is involved in early apoptosis and further induce the proton and other molecules going out of the outer mitochondrial membrane [44,46]. Figure 6 showed that DG suppressed the mitochondrial depolarization of H9c2 in a dose-dependent manner. The results implied that DG could provide anti-apoptotic protection to the cardiomyocytes through inhibiting the opening of mPTP induced by a H/R injury. This is consistent with the previous report that pretreatment of DG inhibited the opening of MPT pores on rat hearts with an I/R challenge [20,21].

Calcium ion stunning also plays a critical role in the initiation of apoptosis in cardiomyocyte in an I/R injury [50]. Our present results revealed that DG treatment greatly inhibited a H/R-induced elevation of cytosolic calcium ion level through living cell recording system under the confocal microscope (Figure 7). The results suggested that the protection by DG may be mediated partly by decreasing the calcium influx into H9c2 cells. As a result, DG may improve the heart function recovery in an I/R Langendorff model by inhibiting the calcium overload within cardiomyocytes. Under an I/R or H/R situation, an ATP lack could induce the sodium overload caused by inhibition of the sarcolemmal Na^+^-K^+^-ATPase and calcium pump [51,52]. The sodium overload would cause calcium overload via increased Na^+^-Ca^2+^ exchange in the reperfusion injury [5]. This revealed the fact that DG may suppress the calcium overload to improve the heart function recovery by protecting the activity of Na^+^-K^+^-ATPase, calcium pump and Na^+^-Ca^2+^ exchanger. Our present result is consistent with a previous report that water extract of Danshen attenuated anoxia and reoxygenation-induced [Ca^2+^_i_ increase in rat cardiac ventricular myocytes and that DG pretreatment decreased the calcium concentration in the rat heart tissue with an I/R injury [53].

A limitation of this study is the use of *ex vivo* model instead of *in vivo* model. The reason is that the animal model of myocardial infarction (MI) which is mimicked by Langendorff heart model, is facing the high mortality rate of the surgical procedure. In brief, after anaesthesia, orotracheal intubation and thoracotomy, the heart is rapidly exteriorised and the coronary artery is ligated in the proximal segment using a thin thread. The occlusion of the artery can be recognised by blanching of the tissue distal to the ligation. In this complicated surgical procedures, a comparative study showed that the mortality of MI in Sprague–Dawley rats was 36% [54]. Therefore, *ex vivo* Langendorff heart model was used in this study instead of *in vivo* model.

## Conclusion

In conclusion, our study firstly demonstrated protection of DG post-treatment against the hypoxic rat cardiomyocytes (H9c2) and ischemic rat hearts from reperfusion injury. The cytoprotection of DG was associated with a decrease in calcium accumulation within H9c2 cells. DG improved H9c2 cell survival, protected the integrity of cell membrane skeleton, decreased the mitochondrial depolarization, decreased proapoptotic and increased anti-apoptotic protein in a dose-dependent manner. In the animal study, DG post-treatment at the reperfusion phase promoted their function recovery and inhibited heart specific enzyme release from a damaged heart. Our results suggested that DG may be a potential supplement for protecting coronary heart disease patients from clinical or spontaneous reperfusion injuries.

## Competing interests

The authors declare that they have no competing interests.

## Authors’ contributions

FH was responsible for performing the experiments, analyzing the data and drafting the manuscript. K-PF, C-MK and JY-WC supervised the whole study and revised the manuscript. All authors have read and approved the final manuscript.

## Pre-publication history

The pre-publication history for this paper can be accessed here:

http://www.biomedcentral.com/1472-6882/12/249/prepub
